# Climate change mitigation and improvement of water quality from the restoration of a subtropical coastal wetland

**DOI:** 10.1002/eap.2620

**Published:** 2022-05-23

**Authors:** Naima Iram, Damien T. Maher, Catherine E. Lovelock, Tallis Baker, Charles Cadier, Maria F. Adame

**Affiliations:** ^1^ Coastal & Marine Research Centre, Australian Rivers Institute Griffith University Nathan Queensland Australia; ^2^ Faculty of Science and Engineering Southern Cross University Lismore New South Wales Australia; ^3^ The School of Biological Sciences The University of Queensland St Lucia Queensland Australia

**Keywords:** floodplain, greenhouse gases, mangroves, *Melaleuca*, methane, nitrogen, nitrous oxide, sugarcane

## Abstract

Coastal wetland restoration is an important activity to achieve greenhouse gas (GHG) reduction targets, improve water quality, and reach the Sustainable Development Goals. However, many uncertainties remain in connection with achieving, measuring, and reporting success from coastal wetland restoration. We measured levels of carbon (C) abatement and nitrogen (N) removal potential of restored coastal wetlands in subtropical Queensland, Australia. The site was originally a supratidal forest composed of *Melaleuca* spp. that was cleared and drained in the 1990s for sugarcane production. In 2010, tidal inundation was reinstated, and a mosaic of coastal vegetation (saltmarshes, mangroves, and supratidal forests) emerged. We measured soil GHG fluxes (CH_4_, N_2_O, CO_2_) and sequestration of organic C in the trees and soil to estimate the net C abatement associated with the reference, converted, and restored sites. To assess the influence of restoration on water quality improvement, we measured denitrification and soil N accumulation. We calculated C abatement of 18.5 Mg CO_2−eq_ ha^−1^ year^−1^ when sugarcane land transitioned to supratidal forests, 11.0 Mg CO_2−eq_ ha^−1^ year^−1^ when the land transitioned to mangroves, and 6.2 Mg CO_2−eq_ ha^−1^ year^−1^ when the land transitioned to saltmarshes. The C abatement was due to tree growth, soil accumulation, and reduced N_2_O emissions due to the cessation of fertilization. Carbon abatement was still positive, even accounting for CH_4_ emissions, which increased in the wetlands due to flooding and N_2_O production due to enhanced levels of denitrification. Coastal wetland restoration in this subtropical setting effectively reduces CO_2_ emissions while providing additional cobenefits, notably water quality improvement.

## INTRODUCTION

Coastal wetlands (mangroves, saltmarshes, and supratidal forests) are important carbon (C) sinks and provide multiple additional ecosystem services, including biodiversity, support for commercial fisheries, coastal protection, and improvements in water quality (Barbier, 2011, pp. 1362–1366; Cheng et al., 2020, p. 625). Because of the multiple benefits, restoration of coastal wetlands contributes toward achieving the Sustainable Development Goals (SDGs) in this Decade of Ecosystem Restoration (Jaramillo et al., 2019, p. 17). However, coastal wetland restoration is a developing field (Bayraktarov et al., 2016, p. 1069), and quantifying the benefits of their restoration is essential to evaluate their contribution in delivering ecosystem services.

Coastal wetland restoration can sequester C through vegetation growth and soil accumulation. Soil C accumulation is favored by anoxic conditions in flooded soils that result in slow organic matter decomposition rates (Mitsch et al., 2013, p. 583). For instance, soil C accumulation in mangroves is 233 ± 280 g C m^−2^ year^−1^ globally; however, this rate is uncertain due to the scarcity of data (Jennerjahn, 2020, p. 9). This is especially true for restoration projects, which are limited, and measurements are usually sporadic or short‐term (Stewart‐Sinclair et al., 2020, pp. 1–2).

Restored coastal wetlands also play an essential role in improving water quality by removing excess nitrogen (N) (Ballantine et al., 2017, pp. 575–576; Cheng et al., 2020, p. 625). Water N as nitrate (NO_3_^−^‐N) can be removed through denitrification, transforming it to N_2_ through a stepwise reduction of NO_2_, NO, and N_2_O as intermediate production. Denitrification is favored in anoxic and C‐rich soils, such as those in coastal wetlands. Thus, soil C will increase as a wetland matures, favoring denitrification and reducing N runoff into adjacent streams (Hey et al., 2012, p. 51). Previous studies focused on landscape‐level modeled effects of restored wetlands to improve water quality (Fennessy & Craft, 2011, p. 49; Jordan et al., 2011, p. 147). However, the scarcity of onsite measurements has resulted in significant knowledge gaps on the benefits of restoring different land‐use types under different management practices, including disused agricultural land (Cheng et al., 2020, pp. 629–630; Peralta et al., 2018, pp. 9–10). Importantly, information on the development of soil denitrification as restoration progresses and, thus, the capacity of the restored wetland to improve water quality is limited (Comer‐Warner et al., 2022, pp. 10–11).

While coastal wetland restoration could increase C storage and provide water quality benefits, restoration usually includes the reinstatement of flooding, for instance, through tidal reconnection. The reinstatement of inundation into former agricultural lands strongly influences the biogeochemistry of soils (Hemes et al., 2018, p. 4107; Marín‐Muñiz et al., 2015, p. 107). For instance, increased denitrification could result in the emission of N_2_O (Davidson & Seitzinger, 2006, p. 2057), which is a potent greenhouse gas (GHG) with a global warming potential 298 times that of CO_2_ (Solomon et al., 2007, p. 33). Given the annual increase in N_2_O globally (Reay et al., 2012, p. 410), emissions from restored wetlands could reduce the climate change mitigation benefits of restoration projects. Still, there are limited data to evaluate this potential tradeoff (Davidson & Seitzinger, 2006, pp. 2060–2061). Furthermore, the anoxic soil conditions that favor denitrification and increase soil C sequestration in freshwater wetlands can also result in significant CH_4_ emissions, another potent GHG (Bridgham et al., 2013, p. 36; Dean et al., 2018, p. 207). Thus, the balance between GHG emissions and C sequestration is critical to understanding the net C abatement of wetland restoration (Mitsch & Mander, 2018, pp. 5–6).

So far, studies on GHG fluxes of restored wetlands have shown conflicting results. Some studies suggest that the production and emissions of CH_4_ from restored wetlands could exceed benefits from soil C storage, turning the system into a net C (Anderson et al., 2016, p. 777; Kandel et al., 2019, p. 527; Rosentreter et al., 2021, pp. 2–3). However, other studies found that restored wetlands have similar emissions to natural wetlands and are lower than those from agricultural land (Audet et al., 2013, p. 170; Morse et al., 2012, p. 264; Tuittila et al., 2000, p. 569). Many of these differences are due to salinity; saline or hypersaline mangroves have low or negligible CH_4_ emissions (Allen et al., 2011, p. 131), whereas freshwater wetlands can be large net sources (Poffenbarger et al., 2011, p. 831). Additionally, mangroves enriched with N are N_2_O sources, while those in N‐depleted waters act as sinks (Maher et al., 2016, p. 1). Many studies have only short‐term measurements of a few land‐use types. Long‐term and in situ monitoring of GHG emissions from restored wetlands from different land uses are needed to understand the local benefits of restored wetlands and their role in the global C cycle (Hemes et al., 2018, p. 4107; Marín‐Muñiz et al., 2015, p. 107).

Accounting for the full suite of benefits of wetland restoration is essential to supporting market schemes such as those for N offsets (e.g., Reef Credits in Australia, de Valck & Rolfe, 2019, pp. 28–29) and climate change mitigation programs. For example, the Emissions Reduction Fund includes methods for reducing GHG emissions by improving land‐use practices in Australia, with an emerging method for coastal wetland restoration with tidal restoration (Kelleway et al., 2020, p. 4; CER, Clean Energy Regulator, 2022). The accurate estimation of C abatement from C sequestration in soils and biomass and avoidance of GHG emissions from prior land use is needed to evaluate the benefits of wetland restoration in helping nations reach their C emission reduction targets (Howard et al., 2014, pp. 17–24).

**FIGURE 1:**
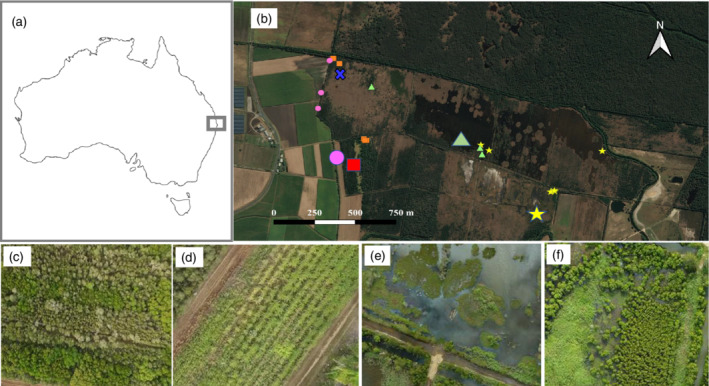
(a) Study area within Maroochy River catchment, Queensland, Australia; (b) location of sampling sites across Yandina Wetlands Restoration Project; (c) reference supratidal forest (*Melaleuca* spp.) (squares in panel b); (d) converted sugarcane (circles); (e) restored saltmarshes (triangles); (f) restored mangroves (stars) and restored supratidal forests (*Melaleuca* spp.) (cross). Large symbols indicate sites for greenhouse gas emission measurements, and smaller symbols are sites for soil and tree sampling.

**FIGURE 2:**
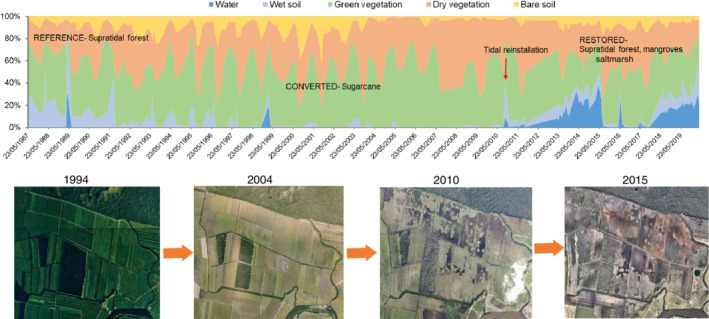
Historical changes in Yandina Wetlands, Queensland, Australia. The site was originally a supratidal forest dominated by *Melaleuca* spp. that was converted to a sugarcane field, and then, following tidal reinstallation in 2010, became a mosaic of supratidal forests, mangroves, and saltmarshes (Wetlandmaps, WetlandInfo, 2021; Google Earth, 2020).

In this study, we measured the climate change mitigation and water quality benefits of restoring a subtropical coastal wetland in Australia. The site was originally a supratidal forest dominated by *Melaleuca* spp. (*reference site*), which was cleared for sugarcane production (*converted site*). After tidal reinstatement, a mosaic of wetlands composed of mangroves, saltmarshes, and supratidal forests (*restored sites*) emerged (Figures 1 and 2). We measured ecosystem functions associated with two ecosystem services: C sequestration and GHG reductions (CO_2_, CH_4_, and N_2_O) for climate regulation and N sequestration and denitrification for water quality improvement (Cadier et al., 2020, p. 8).

We hypothesized that (1) restored wetlands have higher C and N sequestration rates compared to their converted state as a sugarcane field; (2) land‐use type, salinity, and inundation frequency determine the magnitude and direction of GHG fluxes, with N_2_O dominating emissions in the sugarcane field and CH_4_ in the restored wetlands; and (3) the denitrification potential of restored wetlands is higher than the converted sugarcane field but lower than the reference natural wetlands, reflecting soil properties (e.g., soil C, N, redox, C:N). Finally, we present a synthesis of the C abatement of restoring this wetland and a rate of N removal as an indicator of water quality improvement. These results have important implications for supporting beneficial restoration projects that meet expectations for the services they can deliver.

## METHODS

### Site description

The study was undertaken in the Yandina Wetlands Restoration Project within the Maroochy River catchment, Queensland, Australia (Figure 2). The catchment has an area of 63,800 ha and has undergone extensive anthropogenic modification, with ~80% of the total area converted for urban development and agriculture (WetlandInfo, 2021). The site covers 191 ha to the town of Coolum, bordered by Small Creek on the southern side and Yandina Creek on the northern side. There are two main seasons in the area, a dry, cool season (April–September: 23.1 to 26.0°C) and a humid, warm season (October–March: 25.6 to 27.9°C, Australian Bureau of Meteorology, ABM, 2021). The climate is humid subtropical, with a mean annual rainfall of 1706 mm, the highest mean rainfall of 257 mm in February, and the lowest of 54 mm in August (Station 40,157, 1896–2015, ABM, 2021).

Historically, the site was a supratidal forested wetland dominated by *Melaleuca* spp., a wetland often inundated by freshwater but occasionally flooded by tidal water during high spring tides and storm events (Queensland Globe, 2019). In 1864, the sugarcane industry expanded in the area (Australian Cane Farmers Association, 2006), and the wetlands were cleared and drained for farming (Griggs, 2018) (Figure 1), except for a few small forest patches of supratidal forests that remain in the area. In 2005, the local sugar mill closed, and cultivation was abandoned on the site except for in a small area (Figures 1 and 2 Five years later, Unitywater, the local organization responsible for water quality and sewerage management, acquired the land to offset its N inputs to the Maroochy River. Tidal flooding was reinstated in 2010, and wetland vegetation rapidly expanded in the area (Figure 1). Since then, Unitywater has monitored and maintained controlled tidal inundation of the site. The Yandina Wetlands Restoration Project is part of the Blue Heart initiative that aims to restore and protect wetlands in an area of 5000 ha, mainly to manage flooding, but also to improve water quality, enhance C sequestration, increase biodiversity, and provide recreational opportunities.

We used a space‐to‐time approach within a Before‐After‐Control‐Impact framework (BACI design) (Eberhardt, 1976, pp. 30–35; Green, 1979, pp. 181–205) to investigate the development of the ecosystems and the indicators of their services. Our design could not include Before for Control sites, but previous studies showed that studies were robust to this omission (Smokorowski & Randall, 2017, p. 212). We established a control or so‐called reference site, which was the remnant supratidal forest (Figure 1c and Table 1). Our Converted land‐use site (Before site) was a cultivated sugarcane field adjacent to the supratidal forest (Figure 1d and Table 1). The site was harvested from July to December, after which fertilizer was applied at 180 kg N ha^−1^ (farmer personal communication). The sugarcane was rain‐fed, and excess water drained through ditches that flowed to the adjacent Yandina Creek to the Maroochy River. We included a range of habitats within the Restored (After) category: emergent saltmarshes, mangroves, and supratidal forest (*Melaleuca* spp. and *Casuarina glauca*) (Figure 1e, f and Table 1). The sampling was conducted between July 2018 and July 2019.

**TABLE 1:** Locations and characteristics of sites representing trajectory of a restored wetland; the reference is a remnant supratidal forest dominated by *Melaleuca* spp., which was converted to sugarcane, after which tide was reinstated (2010), causing the emergence of saltmarshes, mangroves, and supratidal forests

Site	Description	Latitude	Longitude	Elevation (m)	Dominant species
Reference	Supratidal forest	−26.562	153.037	0.32	*Melaleuca* spp.
Converted	Sugarcane	−26.560	153.034	0.53	*Saccharum officinarum*
Restored	Saltmarshes	−26.563	153.044	0.45	*Sporobolis virginicus*
	Mangroves	−26.565	153.048	0.29	*Avicennia marina*, *Brugiuera gymnorrhiza*, *Exocaria agallocha*
	Supratidal forest	−26.557	153.035	0.40	*Melaleuca* spp., *Acacia* spp., *Casuarina glauca*

*Note*: The elevation is relative to the Australian Height Datum (AHD).

### Carbon and nitrogen stocks

Soil samples were collected in July 2019. We sampled five to eight soil cores along a 50‐m transect from each habitat. The cores were sampled with a 40‐mm‐diameter steel open auger (Dormer, Australia). The sampling depth was targeted at 100 cm; however, it varied for different land‐use types depending on soil density, and cores within the sugarcane land could not be retrieved for depths >30 cm. The core was divided into 5‐ to 10‐cm intervals, transferred to plastic bags, and refrigerated at 4°C until analysis within 1 week of sampling. Redox potential was measured throughout the depth of the core with a redox meter (H.Q. 11d ORP meter, Hach).

Soil samples were oven‐dried at 50°C for 72 h to calculate bulk density by dividing dry weight by volume. Additional subsamples of 5 g were dried to constant weight at 105°C for 48 h to calculate gravimetric soil moisture. Another subsample was ground and homogenized using an electric mortar (RM‐200 Retsch, Hann, Germany) and tested for inorganic C by adding HCL (0.1 M). The addition of HCL did not cause bubbling, indicating that the soil did not contain significant amounts of carbonates. Soil samples were analyzed for organic matter through the loss on ignition method (Heiri et al., 2001, p. 101), and a subset of samples (*n* = 47) was analyzed for %C, %N, δ^13^C, and δ^15^N with an elemental analyzer isotope mass spectrometer (EA‐IRMS, Serco System, Griffith University). We assessed the relationship between organic matter and soil organic carbon (SOC%) for each site to obtain SOC% for the remaining samples.

### Carbon and nitrogen sequestration rates

We measured the biomass of the restored woody forests: mangroves and supratidal forests to estimate aboveground biomass C and N sequestration rates. We used the point‐centered quarter method (Dahdouh‐Guebas & Koedam, 2006) over a 50‐m transect to characterize species composition and estimate aboveground tree biomass from diameter at breast height (DBH) and tree height with allometric equations for each species (Adame et al., 2019, p. 1515; Komiyama et al., 2008, p. 129). Aboveground C stocks were estimated as the biomass multiplied by 0.48 (Kauffman & Donato, 2012, pp. 21–30) and N stocks as the biomass multiplied by 0.001 for the supratidal forests (Adame et al., 2019, pp. 1515–1516) and 0.005 for the mangroves (Gong & Ong, 1990, pp. 520–525). Sequestration rates were estimated as the total biomass and stocks divided by 9 years, the time between the emergence of vegetation and sampling (2010–2019) (Figure 2)

To estimate soil C and N sequestration, we used values from the Tidal Restoration of Blue Carbon Ecosystems Methodology Determination 2021 (Emissions Reduction Fund [ERF], Australian Government), which are derived from published values of sequestration for natural mangroves, saltmarshes, and supratidal forests. Because these values are likely underestimates (Lovelock et al., 2022, p. 4), we calculated an additional upper estimate using the differences in depth of a natural soil horizon. We observed a distinctive gray, mottled clay layer in each sampled core, resulting from marine deposits during the late Quaternary (Roy et al., 1981, p. 471). The layer was found at 42.3 ± 3.4 cm deep in the reference supratidal forest, at 15.3 ± 7.4 cm in the sugarcane, at 31.7 ± 11 cm in the restored supratidal forest, 37.3 ± 7 cm in the restored mangroves, and at 22.7 ± 1 cm in the restored saltmarshes. We estimated the C and N stocks above the marine clay layer for each site, assuming that differences in the soil depth will represent organic matter accumulation due to the wetland productivity over the long term. We subtracted the C stocks from the sugarcane (baseline) from the restored supratidal forest, saltmarsh, and mangrove stocks and divided them by 9 years since vegetation emergence. The difference in the depth of the organic soil layer between the reference supratidal forest and the sugarcane field was 27 cm, which is assumed to be the soil lost during decades of cultivation (~22 Mg C ha^−1^ based on SOC% of remnant supratidal forest, similar to earlier estimates by Hayes et al. [2017], p. 4222; Lovelock et al. [2017], p. 8). We estimated soil N accumulation using the C:N for each ecosystem type and soil depth.

### Soil GHG fluxes

Fluxes of GHG (CO_2_, CH_4,_ N_2_O) were measured every 3 months (2018–2019) at the sugarcane, the reference supratidal forest, the restored saltmarshes, and restored mangroves. Measurements were done with closed chambers (Hutchinson & Mosier, 1981) made of round polyvinyl chloride (PVC) pipes with an internal diameter of 24 cm and a height of 30 cm (base of 18 cm and lid of 12 cm). Five chambers were installed (~3 cm deep) at random locations within each site a day before the experiment to avoid the effects of soil disturbance in gas fluxes. On the day of the experiment, the chambers were closed and sealed tightly using a rubber band. Redox potential was measured next to each chamber using a redox meter (H.Q.11d ORP meter, Hach).

Gas samples were collected from the headspace using a syringe at 0, 20, 40, and 60 min and transferred into vacuumed containers (Labco, High Wycombe, UK) (Brannon et al., 2016). Concentrations of CO_2_, CH_4_, and N_2_O were measured in a gas chromatograph (Micro G.C.), and fluxes were calculated from the linear change in gas concentrations over time corrected by the ideal gas law. Cumulative annual GHG fluxes were calculated by modifying the equation described by Shaaban et al. (2015, p. 19969) as follows:

(1) $$\text{Cumulative annual}\;\mathrm{GHG}\;\text{flux}\;\left(g\;m^{-1}\;{\text{year}}^{-1}\right)=Z_{i=1}\;\left(R_{1}\times 24\;h\times \left(D_{s},+,D_{n}\right)\right)$$

where *R*_*i*_ = gas emission rate (g m^−2^ h^−1^), *D*_*s*_ = number of sampled days, and *D*_*n*_ = number of nonsampled days.

Emissions on nonsampling dates were calculated as the mean of GHG fluxes for two consecutive sampling dates. We calculated CO_2−equivalent_ (CO_2−eq_) of CH_4_ and N_2_O fluxes by multiplying them by their global warming potential of 25 and 29 (Solomon et al., 2007, p. 33). We did not include CO_2_ fluxes in total cumulative annual GHG flux calculations because the chambers were dark; thus, we only accounted for CO_2_ soil respiration and uptake, excluding photosynthesis.

Additionally, we conducted detailed continuous in situ measurements of CO_2_ and CH_4_ in July 2019. We used a Picarro G4301 portable cavity ring‐down spectrometer to measure soil CO_2_ and CH_4_ fluxes with a closed dynamic chamber (Jeffrey, Maher, Tait, & Johnston, 2020, pp. 1678–1679). Briefly, a chamber constructed of PVC pipe (230 mm diameter, 200 mm height) was inserted 2 cm into the sediment. A closed loop between the gas analyzer and chamber was constructed with Bev‐A‐Line IV tubing, with the gas dried through a Drierite column between the outlet of the chamber and the inlet of the analyzer. Gas concentrations were measured at 1 Hz, and the flux was calculated as a function of the surface area, chamber volume, temperature, pressure, and rate of change of gas concentration according to the following formula:

(2) $$\text{Instantaneous}\;\mathrm{GHG}\;\text{flux}\;\left(g\;m^{-2}\;{\mathrm{day}}^{-1}\right)=s\;(V/\left(R\times T\times A\right)$$

where *s* = regression slope (ppm s^−1^), *V* = chamber volume (m^3^), *R* = universal gas constant (8.205 × 10^−5^ m^3^ atm^−1^ K^−1^ mol^−1^), *T* = air temperature inside chamber (°K), and *A* = surface area of chamber (m^2^).

Tree‐stem fluxes of *Melaleuca* spp., *Avicenia marina*, and *Acacia* spp. were measured in a similar way using a modified SNIFF chamber as described by Jeffrey, Maher, Tait, and Johnston (2020, pp. 1678–1679), next to the sampling sites for the soils (Figure 2b).

### Denitrification potential

Denitrification potential was measured with the isotope pairing technique (Nielsen, 1992, p. 357; Steingruber et al., 2001, pp. 3771–3778). This technique enriches the overlying water over sediments with ^15^N‐NO_3_^−^ and estimates ^15^N‐N_2_ gas production with time. The method is based on three assumptions: (1) natural denitrification potential is not affected by ^15^NO_3_^−^ addition, (2) the natural pool of ^14^NO_3_^−^ in sediments and added isotopic ^15^NO_3_^−^ is uniformly mixed, and (3) the isotopic ^15^NO_3_^−^ develops a stable gradient in a short time relative to the incubation period (Robertson et al., 2019, pp. 126–127). To comply with these assumptions, we used the isotopic ^15^NO_3_^−^ concentration equivalent to those measured at the site, ensured a continuous stirring of sediments, and established an incubation period of 5 h, which we have validated to be adequate for tropical wetland soils (Adame et al., 2019, p. 1513).

The day before the experiment, we collected eight soil cores of 10 cm in Perspex tubes (30 cm length and 4.8 cm diameter) per habitat: reference supratidal forest, converted sugarcane, and restored saltmarshes and restored mangroves. The cores contained sediments, fine roots, and overlying litter to simulate natural conditions. They were capped at the base with a rubber bung, filled with water collected from the site, and left overnight to equilibrate. The following day, the experiment was conducted outside with temperature and light conditions like those in the field. The temperature was measured throughout the experiment and kept constant by placing the cores in a plastic container filled with water. Before the experiment, water samples were taken in triplicate from each core to analyze dissolved nutrients. The samples were filtered using a 0.45‐μm membrane filter and stored in the freezer until analysis after a week for NO_x_^−^‐N, ammonium (N‐NH_4_^+^), and phosphate (PO_4_^−^) (calorimetric analyses APHA/AWWA/WPCF, 2012; Chemistry Centre, Department of Science Information Technology and Innovation, Brisbane, Australia).

At the beginning of the experiment, we added 1 ml ^15^N‐KNO_3_^−^ solution (60 μmol L^−1^) to every core. A Teflon‐coated magnetic stirrer bar suspended ~3 cm above the sediments rotated at 60–70 rpm to maintain a homogeneous distribution of ^15^NO_3_^−^ throughout the water column. After ^15^N addition, three water samples were collected from every core to determine ^15^NO_3_^−^ enrichment. Two cores from each site were sacrificed at time 0, three cores at 2 h, and three at 5 h by the addition of 1 ml ZnCl (50% w/v solution) to the overlying water of the core, which was mixed with the sediments with a steel rod to stop the microbial activity. Then three replicate samples of 9 ml were taken from each core using a syringe and transferred into 16‐ml evacuated containers (Labco, High Wycombe, UK) with an additional 250 μl ZnCl (50% w/v solution). The samples were kept refrigerated until analysis within the subsequent 2 weeks.

Headspace gas analyses of ^28^N_2_, ^29^N_2_, and ^30^N_2_ were conducted by extracting 250 μl air from each vacuumed container with airtight syringes injected into a gas chromatograph coupled to an isotope ratio mass spectrometer (EA‐IRMS, Serco System, Griffith University). The detection limit for the denitrification rate was 0.01 mg N m^−2^ h^−1^. We calculated the linear production of ^29^N_2_ (r_29_) and ^30^N_2_ (r_30_) over time using the mean of the three samples per core, which were averaged for the three cores to obtain a mean per site. We used the equations from Steingruber et al. (2001, pp. 3771–3772):

(3) $$D_{15}=r_{29}\times 2\;\left(r_{30}\right),$$

where *D*_15_ is the rate of isotopic ^15^NO_3_^−^ denitrification, *r*_29_ is the production rate of ^29^N_2_, and *r*_30_ is the production rate of ^30^N_2_;

(4) $$D_{14}=D_{15}\times r_{29}/2\;\left(r_{30}\right),$$

where *D*_14_ is the rate of ^14^NO_3_^−^ denitrification;

(5) $$D_{t}=D_{14}+D_{15},$$

where *D*_*t*_ is the total denitrification potential;

(6) $$\varepsilon =({{{\mathrm{NO}}_{3}}^{-}}_{\text{after}}–{\left({{\mathrm{NO}}_{3}}^{-}\right)}_{\text{before}}/{\left({{\mathrm{NO}}_{3}}^{-}\right)}_{\text{after}},$$

where ε is the ^15^NO_3_^−^ enrichment factor;

(7) $${D_{w}}^{t}=D_{15}/\varepsilon ,$$

where *D*_*w*_^*t*^ is the total denitrification potential derived from NO_3_^−^ diffusion through water;

(8) $$D_{w}={D_{w}}^{t}–\left(1-\varepsilon \right),$$

where *D*_*w*_ is denitrification in overlying water without tracer addition;

(9) $$D_{n}=D_{t}-{D_{w}}^{t},$$

where *D*_*n*_ is denitrification coupled with nitrification.

Finally, the denitrification rates measured with the isotope pairing technique were compared to the N_2_O emission obtained with the chamber method (see *Soil GHG fluxes*).

### Statistical analyses

Kolmogorov–Smirnov and Shapiro–Wilk tests were performed to verify the normality of data. When data complied with the assumptions of normality and homogeneity of variances, a one‐way ANOVA test was used to compare differences among sites, with treatment (reference supratidal forest, converted sugarcane, restored supratidal forest, restored saltmarshes, or restored mangroves) as the fixed factor. A repeated‐measurements ANOVA was used to compare differences in GHGs throughout the year, with the month as the fixed factor and chamber as the random factor of the model. When data were not normal, log, inverse, or square root transformations were made to meet the assumptions of normality. Some data were not normal after transformations, so a nonparametric Kruskal–Wallis and Mann–Whitney U tests were performed. We used the statistical program SPSS (version 27, IBM, Armonk, NY) to perform all data analysis. Values are shown as mean ± standard error.

## RESULTS

### Soil characteristics

The reference supratidal forest and the restored wetlands had similar gravimetric soil moisture (*F*_4,55_ = 18.114, *p* > 0.05), which was significantly higher than sugarcane (*F*_4,55_ = 20.92, *p* < 0.05). Gravimetric soil moisture decreased with depth for all sites (*p* < 0.05, Table 2). Redox values were lowest for the reference supratidal forest (−174 ± 25.8 mV) throughout the soil profile and highest in the sugarcane (281.1 ± 32.3 mV). Redox decreased with depth in the reference and restored wetlands, reaching values < −170 mV below 30 cm. The reference supratidal forest, restored mangroves, and restored supratidal forests had lower surface (0–30 cm) soil bulk densities (<1 g cm^−3^) compared to sugarcane and saltmarsh (>1 g cm^−3^). Bulk density increased with depth at all sites (Table 2).

**TABLE 2:** Soil profile characteristics of reference supratidal forest, a sugarcane field, representing converted land, and restored wetlands (saltmarshes, mangroves, and supratidal forests) in Yandina Wetlands, Australia

Site description	Depth (cm)	Water (%)	Redox (mV)	Bulk density (g cm^−3^)	% SOC	% N	δ^13^C (ppm)	δ ^15^N (ppm)	C:N
Reference: Supratidal forest									
	0–15	41.0 ± 1.8	−145.2 ± 18.4	0.44 ± 0.1	33.6 ± 3.8	1.2 ± 0.1	−27.6 ± 0.1	6.7 ± 1.2	37.9 ± 4.6
	15–30	41.5 ± 1.7	−125.0	0.70 ± 0.1	28.5 ± 5.7	1.1 ± 0.1	−27.9 ± 0.0	4.9 ± 1.2	42.4 ± 1.8
	30–50	40.7 ± 0.2	−242.5 ± 2.5	0.98 ± 0.0	9.8 ± 5.7	0.1 ± 0.0	−27.4 ± 0.6	−0.6 ± 0.5	22.9 ± 5.0
	50–100	22.1 ± 2.1	−183.3 ± 46.3	2.4 ± 0.4	1.2 ± 0.9	0.02 ± 0.0	−26.6 ± 0.0	−5.3 ± 2.4	22.9 ± 0.2
Converted: sugarcane									
	0–15	30.5 ± 1.9	248.8 ± 14.2	0.69 ± 0.2	4.9 ± 0.7	0.4 ± 0.0	−23.7 ± 0.6	2.3 ± 0.5	17.4 ± 0.1
	15–30	22.2 ± 2.6	313.3 ± 6.7	1.2 ± 0.1	2.1 ± 0.7	0.1 ± 0.0	−27.2 ± 0.4	0.1 ± 0.2	23.2 ± 0.9
Restored: saltmarshes									
	0–15	39.4 ± 1.6	−64.7 ± 34.8	0.9 ± 0.1	5.5 ± 0.3	0.3 ± 0.0	−25.8 ± 0.4	0.6 ± 0.2	22.3 ± 0.7
	15–30	31.8 ± 3.4	−155.0 ± 42.5	1.2 ± 0.1	2.5 ± 0.4	0.9 ± 0.8	−27.1 ± 0.2	4.9 ± 5.1	29.3 ± 1.3
	30–50	28.5 ± 3.4	−197.5 ± 42.5	1.4 ± 0.1	1.9 ± 0.3	…	…	…	…
	50–100	39.8 ± 1.2	−174.2 ± 30.8	1.1 ± 0.0	2.9 ± 0.3	…	…	…	…
Restored: mangroves									
	0–15	48.6 ± 1.6	−100.2 ± 40.2	0.6 ± 0.0	4.9 ± 0.3	0.4 ± 0.0	−25.4 ± 1.0	3.8 ± 0.5	18.1 ± 0.7
	15–30	42.1 ± 3.2	−94.4 ± 46.5	1.0 ± 0.1	3.7 ± 0.3	0.3 ± 0.0	−26.6 ± 0.5	1.6 ± 0.9	19.1 ± 0.2
	30–50	43.4 ± 4.4	−170.8 ± 9.2	1.6 ± 0.1	3.6 ± 0.8	0.2 ± 0.2	−26.0 ± 0.4	−1.0 ± 3.6	29.7 ± 12.1
	50–100	42.5 ± 1.5	−192.4 ± 14.9	1.9 ± 0.1	4.9 ± 0.9	0.2 ± 0.1	−27.1 ± 0.1	1.6 ± 2.2	22.4 ± 4.2
Restored: supratidal forest									
	0–15	45.2 ± 3.1	−142.5 ± 4.2	0.4 ± 0.8	7.4 ± 0.8	0.8 ± 0.4	−24.8 ± 1.3	5.9 ± 4.6	24.0 ± 4.0
	15–30	38.3 ± 0.3	−180.0	0.7	5.9 ± 0.9	0.3 ± 0.0	−26.6 ± 0.5	0.3 ± 0.3	22.4 ± 1.7
	30–50	34.9 ± 3.4	−215.5 ± 32.5	1.0	4.6 ± 0.9	0.2 ± 0.1	−28.2 ± 0.4	−0.2 ± 0.2	27.0 ± 1.2
	50–100	…	…	2.1	3.4 ± 2.6	…	…	…	…

*Note*: Values are mean ± standard error (*n* = 5–8 cores per habitat).

Abbreviations: N, nitrogen; SOC, soil organic carbon.

### C and N stocks and sequestration rates

All sites had significantly different soil SOC (*F*_4,55_ = 65.26, *p* < 0.05), with the reference supratidal forests having the highest (mean of sediment column of 18.1% ± 7.5%) and the sugarcane and restored saltmarshes the lowest (3.5% ± 1.4% and 3.2% ± 0.8%, respectively) (Table 2). Within the soil profile, SOC decreased with depth at all sites except in the restored mangroves (Table 2). For soil N, the highest values were found in the reference supratidal forests (0.63% ± 0.32%) and restored saltmarshes (0.63% ± 0.30%) and lowest in the sugarcane (0.24% ± 0.14%) and restored mangroves (0.25% ± 0.03%). Soil N also tended to decrease with depth (Table 2).

Soil C:N ratios also varied significantly among sites (*F*_4,20_ = 17.11, *p* < 0.05), with the highest values for the reference supratidal forest (31.5 ± 5.1), followed by restored saltmarshes (25.8 ± 3.5), restored supratidal forest (24.5 ± 1.4), and restored mangroves (22.3 ± 2.6), and the lowest values in sugarcane (20.3 ± 2.9) (Table 2). For the reference supratidal forest, soil C:N ratio decreased with depth, but for sugarcane and restored saltmarshes and mangroves, the C:N increased (*t* = 7.98, *n* = 10, *p* < 0.05; *t* = 19.0, *n* = 10, *p* < 0.05, *t* = 19.0, *n* = 20, *p* < 0.05, respectively).

Soil stocks were 1180 ± 271 Mg C ha^−1^ and 34.7 ± 1.2 Mg N ha^−1^ for the reference supratidal forest (100 cm), 528 ± 136 Mg C ha^−1^ and 30.3 ± 18.9 Mg N ha^−1^ for the restored mangroves (100 cm), 399 ± 6 Mg C ha^−1^ and 15.6 ± 0.4 Mg N ha^−1^ for the restored saltmarshes (100 cm), and 250 ± 31 Mg C ha^−1^ and 9.4 ± 1.2 Mg N ha^−1^ for the restored supratidal forest (50 cm). The lowest stocks were measured in the sugarcane with 76 ± 22 Mg C ha^−1^ and 5.2 ± 1.2 Mg N ha^−1^ (30 cm). The C sequestration rates from tree growth (1–3.5 Mg C ha^−1^ year^−1^) were higher than those from the soils, as reported for the Australian Blue Carbon method (0.6–1.4 Mg C ha^−1^ year^−1,^ Clean Energy Regulator (CER), 2022) but lower when considering the upper estimations derived from the marine clay observed in the cores (8–10.6 Mg C ha^−1^ year^−1^) (Table 3).

**TABLE 3:** Forest structure, carbon (C) and nitrogen (N) stocks, and sequestration rates for restored supratidal forests, mangroves, and saltmarshes, 9 years since vegetation emerged (2010–2019) in Yandina Wetlands, Queensland, Australia

Parameter	Unit	Restored supratidal forest	Restored mangroves	Restored saltmarshes
Tree density	trees ha^−1^	6283	2061	…
Tree height	m	7.0 ± 0.3	3.2 ± 0.7	…
DBH	cm	6.9 ± 0.4	4.7 ± 0.3	…
AGB	Mg ha^−1^	64.8	19.0	n.a
AGB accumulation	Mg ha^−1^ year^−1^	7.2	2.1	n.a
AGB stock	Mg C ha^−1^	31.1	9.1	n.a.
	Mg N ha^−1^	0.06	0.05	n.a.
AGB accumulation	Mg C ha^−1^ year^−1^	3.5	1.0	n.a.
	Mg N ha^−1^ year^−1^	0.01	0.01	
Soil C stock to 1 m	Mg C ha^−1^	415^a^	528	399
Soil N stock to 1 m	Mg N ha^−1^	30.3^a^	15.0	15.6
Soil C sequestration	Mg C ha^−1^ year^−1^	0.62 (8.0)	1.40 (10.6)	0.77 (3.5)
	Mg N ha^−1^ year^−1^	0.03 (0.4)	0.18 (1.0)	0.03 (0.1)
Soil accumulation above sugarcane baseline	cm year^−1^	1.1	1.5	0.4

*Notes*: Soil C sequestration is from Australian Blue Carbon Method (CER, Australian Government). An upper estimate of soil C and N sequestration (in parentheses) was calculated from the variation in organic soil depth to a marine clay layer in relation to the sugarcane baseline.

Abbreviations: AGB, aboveground biomass; DBH, diameter at breast height (cm); n.a., no data available.

^a^ Extrapolated from 50 to 100 cm.

### Soil GHG fluxes

**FIGURE 3:**
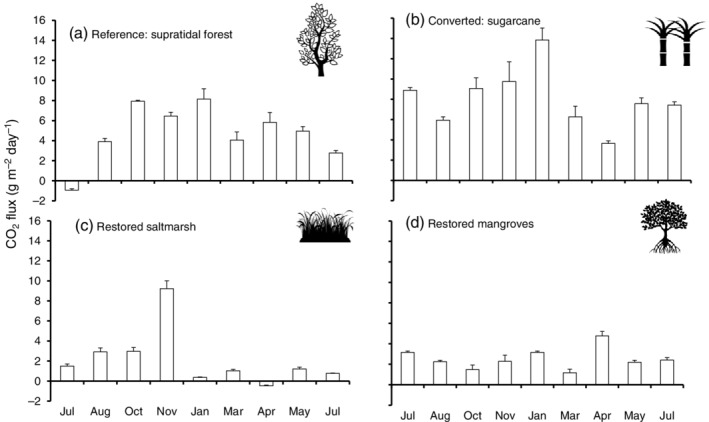
Annual CO_2_ fluxes (g m^−2^ day^−1^, July 2018–July 2019) in reference supratidal forest, (b) sugarcane, and restored (c) saltmarshes and (d) mangroves. (Icons from B. Mania, A. Santos, ardiezt and Richard, The Noun Project)

Soil CO_2_ emissions varied among sites (*F*_24,144_ = 1.395, *p* < 0.05) (Figure 3a–d). Emissions from sugarcane were higher than those of wetlands throughout the study period, with the highest emissions in January (wet‐hot season) when they peaked at 13.8 ± 1.2 g CO_2_ m^−2^ day^−1^ (Figure 3b). The reference supratidal forest had fluxes between −0.9 ± 0.1 and 8.2 ± 1.0 g CO_2_ m^−2^ day^−1^, which differed significantly (*p* < 0.05) across the annual cycle, with the lowest values during the dry‐cold season (July 2019) when the site was a minor sink (−0.9 ± 0.1 g CO_2_ m^−2^ day^−1^) (Figure 3a). The restored saltmarshes had low CO_2_ emissions for most of the year, except in November, when emissions reached 9.2 ± 0.8 g CO_2_ m^−2^ day^−1^ (Figure 3c). For restored mangroves, CO_2_ emissions were similar throughout the year (*p* > 0.05) (Figure 3d).

**FIGURE 4:**
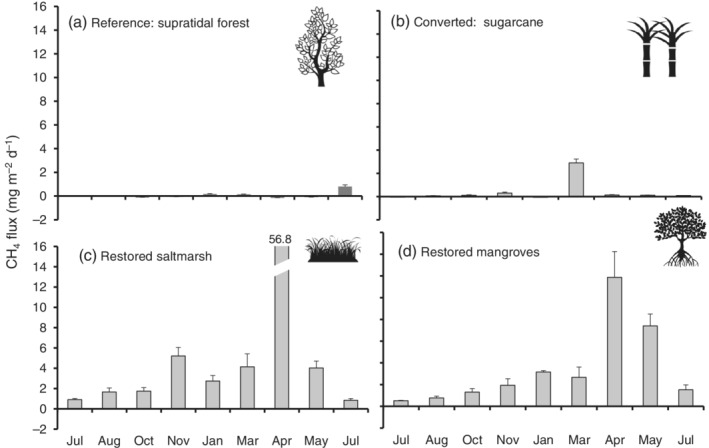
Annual CH_4_ fluxes (mg m^−2^ day^−1^, July 2018–July 2019) in reference supratidal forest, (b) sugarcane, and restored (c) saltmarshes and (d) mangroves. (Icons from B. Mania, A. Santos, ardiezt and Richard, The Noun Project)

The CH_4_ fluxes varied significantly among sites (*t* = 104.857, *n* = 180, *p* < 0.05) (Figure 4). Emissions from the reference supratidal forest (−0.2 to 2 mg CH_4_ m^−2^ day^−1^) and sugarcane (−0.1 to 2.9 mg m^−2^ day^−1^) were similar (*p* = 0.16) (Figure 4a,b) and lower than those of restored saltmarshes and mangroves (0.8 to 12 mg m^−2^ day^−1^; *p* = 0.73) (Figure 4c,d). On one occasion, the sugarcane had a spike of CH_4_ during March, the hot‐wet season (Figure 4b). The highest CH_4_ emissions of 57 ± 18 mg m^−2^ day^−1^ and 12 ± 2 mg m^−2^ day^−1^ were recorded during April in the restored saltmarshes and mangroves, respectively, at the end of the hot‐wet season (Figure 4c,d).

**FIGURE 5:**
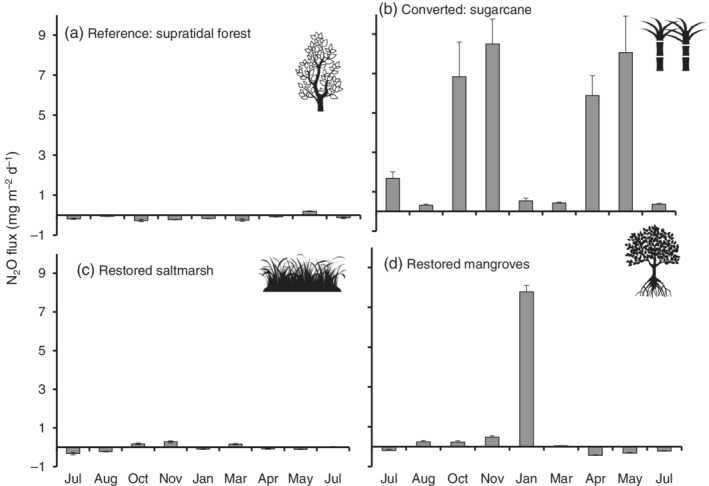
Annual N_2_O fluxes (mg m^−2^ day^−1^, July 2018–July 2019) in reference supratidal forest, (b) sugarcane, and restored (c) saltmarshes and (d) mangroves. (Icons from B. Mania, A. Santos, ardiezt and Richard, The Noun Project)

The N_2_O emissions from sugarcane (*p* < 0.05) were one order of magnitude higher than those for wetlands, with values of over 8 mg m^−2^ day^−1^ measured in November and May (Figure 5b). In contrast, the reference supratidal forest was consistently a N_2_O sink throughout the year, except during May (dry‐cold season), when the site was a minor source with 0.2 ± 0.0 mg N_2_O m^−2^ day^−1^ (Figure 5a). The restored saltmarshes were either a sink or a small N_2_O source, with values ranging from −0.2 to 0.2 mg N_2_O m^−2^ day^−1^ (Figure 5c). Finally, the restored mangroves were also either a sink (−0.4 ± 0 mg N_2_O m^−2^ day^−1^) or a small source (0.5 ± 0.1 mg N_2_O m^−2^ day^−1^), except for one peak measured during the hot season (Jan), when emissions reached 7.8 mg m^−2^ day^−1^ (Figure 5d).

### Cumulative annual GHG soil fluxes

The highest cumulative annual CO_2_ emissions were measured in the sugarcane with 30.8 Mg ha^−1^ year^−1^, followed by the reference supratidal forest with 19.9 Mg ha^−1^ year^−1^ and restored saltmarshes and mangroves with 8.5 and 8.9 Mg ha^−1^ year^−1^, respectively. For CH_4_, the restored saltmarshes and mangroves had the highest emissions at 2.8 × 10^−2^ Mg ha^−1^ year^−1^ and 1.2 × 10^−2^ Mg ha^−1^ year^−1^, respectively, followed by sugarcane (1.6 × 10^−3^ Mg ha^−1^ year^−1^) and the reference supratidal forest (2.6 × 10^−4^ Mg ha^−1^ year^−1^). For N_2_O emissions, the sugarcane was the highest emitter with 1.4 × 10^−2^ Mg ha^−1^ year^−1^, followed by the restored mangroves with 4.8 × 10^−3^ Mg ha^−1^ year^−1^. In contrast, the reference supratidal forest and the restored saltmarshes were sinks of N_2_O with −0.46 × 10^−4^ and − 8.6 × 10^−6^ Mg ha^−1^ year^−1^, respectively.

Overall, considering CO_2−eq_ values for CH_4_ and N_2_O emissions together, the sugarcane had the highest emissions at 4.1 Mg CO_2−eq_ ha^−1^ year^−1^, followed by restored mangroves and saltmarshes at 1.7 and 0.7 Mg CO_2−eq_ ha^−1^ year^−1^, respectively. The reference supratidal forest was a sink with −0.1 Mg CO_2−eq_ ha^−1^ year^−1^.

### Instantaneous water, soil, and tree GHG

The instantaneous measurements of GHG (CH_4_ and CO_2_) were within the same range of the annual values recorded for the restored mangroves, restored saltmarshes, and sugarcane. However, in the reference supratidal forest, the instantaneous measurements were higher, and tree instantaneous measurements were also high for *Melaleuca* stems (Table 4).

**TABLE 4:** Instantaneous CH_4_ and CO_2_ emissions from soil, water, and trees measured 20–22 July 2019 in Yandina Wetlands

Substrate	Type	CH_4_ (mg m^−^^2^ day^−1^)	CO_2_ (g m^−^^2^ day^−1^)
Water	Floodgate/ditches	1.36 ± 0.29	10.93 ± 3.74
	Open water	1.61 ± 0.18	2.30 ± 0.17
Soil	Supratidal forest	15.11 ± 5.12	15.10 ± 3.86
	Mangroves/rush	1.59 ± 0.82	12.34 ± 0.02
	Marsh	0.37 ± 0.17	14.80 ± 1.71
	Sugarcane	0.14 ± 0.14	12.85 ± 10.68
Trees^a^	*Melaleuca* spp.	34.34 ± 24.21	12.07 ± 3.54
	*Casuarina*	0.43 ± 0.15	32.58 ± 7.54
	Mangrove	2.90 ± 2.31	17.10 ± 6.61

^a^ Emissions per area of stem.

### Denitrification potential

**FIGURE 6:**
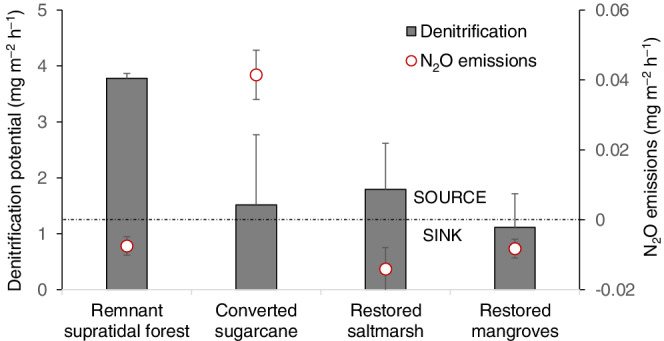
Denitrification potential (*D*_*t*_) versus N_2_O fluxes in reference supratidal forest, a sugarcane field, and restored saltmarshes and mangroves. The dashed line indicates whether the site was a sink or a source of N_2_O

The reference supratidal forest had the highest denitrification potential (*D*_tot_) with 3.8 ± 0.1 mg N m^−2^ h^−1^, most of which was coupled with nitrification (*D*_*n*_). The sugarcane soil and the restored wetlands had lower and similar denitrification values ranging between 1.8 and 2.8 mg N m^−2^ h^−1^, with over half of the denitrification fueled by NO_3_^−^ from the overlying water (*D*_*w*_^tot^) (Table 5). Contrary to the patterns of denitrification, the highest N_2_O emissions were measured in the sugarcane, suggesting that nitrification and incomplete denitrification were the main N pathways at this site. The reference and restored wetland soils were sinks of N_2_O, despite having high denitrification potential. This result suggests that in these wetlands, denitrification was complete, generating N_2_ instead of N_2_O (Figure 6).

**TABLE 5:** Nutrient concentration during experiment and denitrification rates (mg m^−2^ h^−1^) of reference supratidal forest, a sugarcane field, and restored saltmarshes and mangroves

Site	Nutrient concentration (mg L^−1^)				Denitrification rate (mg m^−2^ h^−1^)			
	N‐NH_4_^+^	N‐NO_*x*_^−^	PO_4_^−^	ε	*D*_tot_	*D*_*w*_^tot^	*D*_*w*_	*D*_*n*_
Reference supratidal	0.38 ± 0.01	0.03 ± 0.00	0.004 ± 0.002	0.17	3.8 ± 0.1	0.2 ± 0.0	0.2 ± 0.0	3.1 ± 0.1
Converted sugarcane	0.15 ± 00.4	0.01 ± 0.00	0.009 ± 0.001	0.61	2.8 ± 0.5	1.4 ± 0.3	0.6 ± 0.1	0.9 ± 0.2
Restored marsh	0.10 ± 0.03	0.01 ± 0.00	0.001 ± 0.000	0.63	1.8 ± 0.8	1.2 ± 0.5	0.4 ± 0.2	0.6 ± 0.3
Restored mangroves	0.07 ± 0.00	0.01 ± 0.00	0.009 ± 0.000	0.60	2.1 ± 0.2	1.2 ± 0.1	0.5 ± 0.1	0.8 ± 0.1

Abbreviations: ε, NO_3_^−^ enrichment factor during experiment; *D*_*n*_, coupled nitrification–denitrification; *D*_*t*_, total denitrification; *D*_*w*_^tot^, total denitrification of labeled plus unlabeled NO_3_^−^ from water column; *D*_*w*_, natural denitrification rate without tracer addition.

## DISCUSSION

Nine years after tidal reintroduction, the Yandina Wetlands are providing significant GHG mitigation through woody vegetation growth, soil accretion, and reduction of GHG emissions. Additionally, the restored wetlands provide water quality improvement by developing C‐rich and anaerobic soils, favoring denitrification.

The restored mangroves and supratidal forests are accumulating wood biomass at 2.1 and 7.2 Mg ha^−1^ year^−1^, respectively. For the mangroves, the values are within the global range of natural forests of 0.3 to 45.5 Mg ha^−1^ year^−1^ and those in Australia (0.8 to 45.1 Mg ha^−1^ year^−1^) (Xiong et al., 2019, p. 387), which are similar to those for restored mangroves (Sasmito et al., 2020, p. 3028). For the restored supratidal forests, the wood biomass accumulation rates are like mature forests in tropical Australia with 10.4 ± 4.2 Mg ha^−1^ year^−1^ (Adame et al., 2020, p. 454). Our values did not include large roots for the belowground biomass and so likely underestimate C sequestration by the trees.

The upper rates of soil C sequestration using depth of soil to the marine clay horizon (3.5–10.6 Mg C ha^−1^ year^−1^) were 10‐fold higher than those from the Australian Blue Carbon Method (CER, 2022) and other restored mangroves (1.9–2.4 Mg C ha^−1^ year^−1^) (Duarte de Paula Costa et al., 2021, pp. 3261–3266). However, the estimated upper values were within the range reported for tropical mangroves in Malaysia (Adame et al., 2018, p. 117) and those for the first few years after tidal reintroduction for salt ponds in South Australia (Jones et al., 2019, pp. 7–9) (Jones et al., 2019) and after reflooding of saltmarshes in the UK (Dale et al., 2019, pp. 1419–1420). Measuring accumulation over a horizon may tend to overestimate C sequestration (Lovelock et al., 2017, p. 8; Lovelock et al., 2022, p. 4), and our measurements over the natural horizon have assumptions and high uncertainty levels. However, soil C accumulation may be rapid in the initial stages of reflooding sites but particularly low in the intertidal zone. Measurements of soil C sequestration through monitoring changes in soil volume against a known benchmark using surface elevation tables will help tackle these uncertainties in the future (Howard et al., 2014, pp. 17–24).

Besides the increase in C/N stocks with age, we found that the changes in land use had a significant impact on GHG fluxes, as observed globally (Table 6). As hypothesized, N_2_O emissions were highest in the sugarcane compared to the wetlands, and in fact, the reference supratidal forest and restored saltmarshes were regular N_2_O sinks. The N_2_O emissions peaked in the sugarcane in October–November and April–May, and there was a one‐off peak for the mangroves in January. The sugarcane also had the highest CO_2_ emissions compared to any other site. These results were expected because N_2_O is directly associated with fertilization and CO_2_ to respiration of dry soils (Rastogi et al., 2002, p. 513).

**TABLE 6:** Total annual cumulative greenhouse gas (GHG) (CH_4_ + N_2_O, CO_2−eq_ Mg ha^−2^ year^−1^) fluxes in natural, agriculturally converted, and restored wetlands in (sub)tropical climates

Country	Agricultural lands	Natural mangroves	Natural saltmarshes	Forested wetlands	Restored wetlands	Reference
USA	0.9 to 42.1	…	…	−0.5 to 9.1	−0.04 to 18.0	Morse et al. (2012)
USA		…	…	−0.7 to 114.0	…	Yu et al. (2008)
China	0.2 to 0.9	…	…	…	…	Liu et al. (2008)
Global	…	−0.9 to 113.5	−8.0 to 17.8	…	…	Al‐Haj and Fulweiler (2020)
Global	…	−1.9 to 6.0	9.8 to 20.7	…	…	Rosentreter et al. (2021)
Australia	…	−0.4	…	…	…	Maher et al. (2018)
Australia	7.1	−0.2	−1.0	−0.2	…	Iram et al. (2021)
Australia	4.1	…	…	−0.1	−0.7 to −1.8	This study

*Note*: Positive values are emissions, negative values are uptake.

In contrast, CH_4_ emissions were highest in the restored saltmarshes, especially during April, followed by the mangroves. The reference supratidal forest soil had very low CH_4_ emissions, even though it was freshwater and had high SOC. We have described similar results in Northern Australia (Iram et al., 2021, p. 5089), which can be partially attributed to *Melaleuca* emitting CH_4_ from the stems of the trees (Jeffrey, Maher, Tait, Euler, et al., 2020, p. 273) and probably due to soil microbiomes that can potentially oxidize CH_4_ decreasing emissions (Jeffrey et al., 2021, p. 1).

We measured significant changes in the soil properties when comparing the reference, converted, and restored wetlands. The reference supratidal forest had the lowest redox (mV), high water content, lowest bulk density, and lowest δ^13^C values; it also had the highest soil %C, highest C:N, highest surface δ^15^N, and highest denitrification potential. In contrast, the sugarcane had the lowest soil water content, lowest C:N, and lowest surface δ^15^N; it also had the highest redox and highest soil δ^13^C values. The restored wetlands had intermediate values. These changes in soil conditions are associated with increased productivity (high soil C and C:N), changes in organic C sources (low values due to transition from C_4_ to C_3_ plants), increased denitrification potential, and decreases in fertilization (higher δ^15^N values) and flooding (low redox and high water content). These relatively simple soil parameters could be used as indicators of the progress of wetland restoration projects (Cadier et al., 2020, p. 8).

## IMPLICATIONS FOR NATIONAL CARBON SCHEMES

Australia has committed in the Paris Agreement to reduce its emissions by 26%–28% from 2005 levels by 2030. A large portion of this is expected to be achieved through improved land‐use practices. This study shows that restoring marginal or unproductive sugarcane fields can achieve some of these CO_2_ mitigation targets. During the first years of restoration, the highest reductions would be achieved by reducing GHG emissions through stopping fertilisation. Longer‐term reductions would be achieved by tree and soil accumulation. Together these processes can offset the CH_4_ emissions triggered by land flooding. The Yandina Wetlands are a pilot study for future restoration projects in Australia. Here we have shown that their restoration is mitigating between 6.2 and 18.5 Mg CO_2−eq_ ha^−1^ year^−1^ depending on the vegetation type established (Table 7) while also providing the cobenefit of water quality improvement. Other cobenefits, such as green space amenities and biodiversity values, are currently being explored and will provide evidence for further ecosystem benefits that restoring coastal wetlands can provide.

**TABLE 7:** Total carbon mitigation (Mg CO_2−eq_ ha^−1^ year^−1^) from restoring sugarcane land into coastal wetlands (supratidal forests, saltmarshes, and mangroves)

Conversion of sugarcane	Soil GHG reduction	Soil sequestration	Tree growth	Tree emissions	Total mitigation
To supratidal forest	4.20	2.3	12.7	−0.63	18.5
To mangroves	2.33	5.2	3.7	−0.26	11.0
To saltmarshes	3.38	2.8	…	…	6.2

*Notes*: GHG emissions are calculated as those from sugarcane minus those from restored wetlands. Tree emissions of *Melaleuca* spp. are from Jeffrey, Maher, Tait, and Johnston (2020), soil sequestration rates are from CER (2022), and mangrove tree emissions are estimates from one sampling in July 2019. CO_2_‐equivalent (CO_2_‐eq) of CH_4_ and N_2_O fluxes were obtained by multiplying them by their global warming potential of 25 and 298.

Abbreviations: GHG, greenhouse gas.

## AUTHOR CONTRIBUTIONS

Naima Iram, Catherine E. Lovelock, and Maria F. Adame designed the project. Naima Iram, Tallis Baker, and Charles Cadier carried out the experiments. Naima Iram, Damien T. Maher, and Catherine E. Lovelock analyzed the data. Naima Iram and Maria F. Adame prepared the manuscript, with contributions from Damien T. Maher, Catherine E. Lovelock, Tallis Baker, and Charles Cadier.

## CONFLICT OF INTEREST

The authors declare no conflict of interest.

## Data Availability

Data (Adame et al., 2022) are available in the Griffith University Research Data Repository at: https://doi.org/10.25904/1912/4432.
